# Factors That Determine the Dietary Diversity Score in Rural Households: The Case of the Paute River Basin of Azuay Province, Ecuador

**DOI:** 10.3390/ijerph18042059

**Published:** 2021-02-20

**Authors:** Otilia Vanessa Cordero-Ahiman, Jorge Leonardo Vanegas, Christian Franco-Crespo, Pablo Beltrán-Romero, María Elena Quinde-Lituma

**Affiliations:** 1 Grupo de Investigación en Economía Regional (GIER), Facultad de Ciencias Económicas y Administrativas, Universidad de Cuenca, Cuenca 010107, Ecuador; otilia.cordero@ucuenca.edu.ec (O.V.C.-A.); pablo.beltran@ucuenca.edu.ec (P.B.-R.); mariae.quinde@ucuenca.edu.ec (M.E.Q.-L.); 2 Grupo de Producción Animal e Industrialización (PROANIN), Facultad de Ingeniería, Universidad Nacional de Chimborazo, Riobamba 060103, Ecuador; 3 Facultad de Ciencia e Ingeniería en Alimentos y Biotecnología, Campus Huachi, Universidad Técnica de Ambato, Ambato 180104, Ecuador; franco.crespo.ec@gmail.com

**Keywords:** dietary diversity, HDDS, Poisson regression model, rural area, Ecuador

## Abstract

Inadequate food and nutrition affect human well-being, particularly for many poor subpopulations living in rural areas. The purpose of this research was to analyze the factors that determine the Household Dietary Diversity Score (HDDS) in the rural area of the Paute River Basin, Azuay Province, Ecuador. The sample size of 383 surveys was determined by a stratified random sampling method with proportional affixation. Dietary diversity was measured through the HDDS, with 12 food groups (cereals; roots and tubers; fruits; sugar/honey; meat and eggs; legumes or grains; vegetables; oils/fats; milk and dairy products; meats; miscellaneous; fish and shellfish) over a recall period of 7 days. A Poisson regression model was used to determine the relationship between the HDDS and sociodemographic variables. The results show that the average HDDS of food consumption is 10.89 foods. Of the analyzed food groups, the most consumed are cereals; roots and tubers; fruits; sugar/honey. In addition, the determinants that best explain the HDDS in the predictive model were housing size, household size, per capita food expenditure, area of cultivated land, level of education, and marital status of the head of household. The tools used in this research can be used to analyze food and nutrition security interventions. Furthermore, the results allow policymakers to identify applicable public policies in the fight against hunger.

## 1. Introduction

A United Nations Food and Agriculture Organization (FAO) report [1] indicates that in 2019, about 690 million people around the world were malnourished. This situation can be aggravated in developing countries and especially in rural sectors, which show the highest concentration of poor people. The causes of malnourishment are varied, but it occurs more where there is evidence of an increase in food insecurity, often related to the presence of climate change phenomena. In 2018, Latin America (LA) experienced an increase in the number of people living in a situation of hunger, reaching 42.5 million people [2], a number that may be even higher in 2020 due to the contraction of the economy caused by the COVID-19 pandemic. In the region of LA, post-COVID-19 projections indicate a decrease in the well-being of the population. Unemployment has seen a significant impact, going from 8.1% (2019) to 13.5% (2020), and the share of the population living in poverty may reach 37.2%, and extreme poverty 15.5% [3]. A decrease in the ability of households to access healthy and nutritious food is thus expected, due to a reduction in family income [4].

Access to sufficient and nutritious food is a crucial factor for reducing food insecurity [5,6]. Efforts to ensure food security are related to socioeconomic factors and the level of information available regarding a healthy and balanced diet [7,8]. Particular sociodemographic factors are also related to the amount of consumption of foods such as fruits, vegetables and proteins, the consumption of which is related to the prevention of adverse health conditions [8]. Moreover, it has been identified that food consumption patterns outside the home can affect the dietary diversity of families [9].

The negative effects of a poor diet can seriously affect children from 0 to 5 years old and adults over 60 years of age. A lack of proper nutrition can cause poor growth in children and reduced quality of life in adults [10]. Effectively addressing malnutrition and nutrient deficiencies in the rural sector remains a critical international priority, especially as rural populations are the most affected by this double burden of undernutrition [11].

In rural areas, food is based on existing resources in the environment and the ability to obtain food through agricultural production [5]. More specifically, in agricultural production areas the availability of a diversity of agricultural products for self-consumption increases the quality of a family’s diet [5]. Reducing farmer’s obstacles to access to markets promotes dietary diversity in households [12,13]. Thus, the appropriate development of good practices for reducing malnutrition depends on the design and implementation of public policies with attention to vulnerable groups and women. The guarantee of access to water and environment of good nutrition could be the key for improvement of diverse food [14].

Dietary diversity (DD), especially between and within food groups and between different varieties of specific foods, is vital for a high-quality diet as it more or less guarantees an adequate intake of essential nutrients and important non-nutritive factors [15]. DD is measured by counting the number of different foods or food groups in a diet. However, a number of different groups, classification systems, and reference periods have been used [16,17]. Despite the multiple approaches used to measure dietary diversity and the varying determinants of diversity across locations, findings from multiple contexts consistently confirm the importance of including a diverse selection of foods into one’s diet [15]. DD is affected by lack of income, mainly as a result of the lower consumption of proteins, fruits and vegetables. Protein-rich foods, as well as those considered healthy, are more expensive than foods high in carbohydrates or saturated fat [18].

Household dietary diversity (HDD) is an instrument for measuring the economic capacity of a household to access a variety of foods during a given period [19]. The dietary diversity questionnaire described by Kennedy [20], which is used to create the Household Dietary Diversity Score (HDDS), is an easily applicable tool to assess access to food and is widely used to qualitatively determine food consumption, including the level of variety of foods a household has access to [21,22]. Similar studies show that the diversity of agricultural production is positively associated with DD, although access to markets has an even greater impact on dietary diversification [23]. In addition, socioeconomic factors such as level of education, income and information on healthy eating also have a significant influence on DD [24].

The evaluation of farmer HDD has focused on the contribution of food grown for self-consumption [21]. According to Jones et al. [14], the availability of foods that facilitate their access increases the diversity of the diet. Alam [12], for his part, mentions that access to information is a factor that affects decision-making to achieve a nutritional balance. In addition, enrollment in informed groups creates a conducive environment to positive changes in eating behavior [8].

In rural Ecuador, factors that can increase food security include the number of household members and information on the benefits of a good diet, i.e., safe and diversified food [25]. In areas inhabited by Indigenous communities, lack of irrigation as well as the loss of traditions regarding agricultural production and food culture are identified as the main causes of poor nutrition [26]. Market imperfection reduces the ability of Indigenous communities to produce food for self-consumption or to supply it to the market [27]. The availability of local foods influences the diversification of macro- and micronutrients in the diets of rural populations [28]. However, more research is needed to better understand nutritional and dietary outcomes and to determine the best ways to measure the impact of agriculture on nutrition and dietary outcomes [29].

This research aims to analyze the factors that determine the Household Dietary Diversity Score (HDDS) in the rural area of the Paute River Basin in Azuay Province, Ecuador, to enable policymakers to identify the most effective strategies to apply in the fight against hunger. After this Introduction, the second section describes the empirical materials and methods used in this study. The third section presents the results of a statistical and econometric analysis. The fourth section offers a discussion of the results, and the last section offers some concluding remarks.

## 2. Materials and Methods

This section describes the study area, sample design, data collection, and dietary diversity measurement (the indicator of dietary diversity), as well as the Poisson regression model implemented to determine relationships between variables.

### 2.1. Location of the Study Area

**Figure 1:**
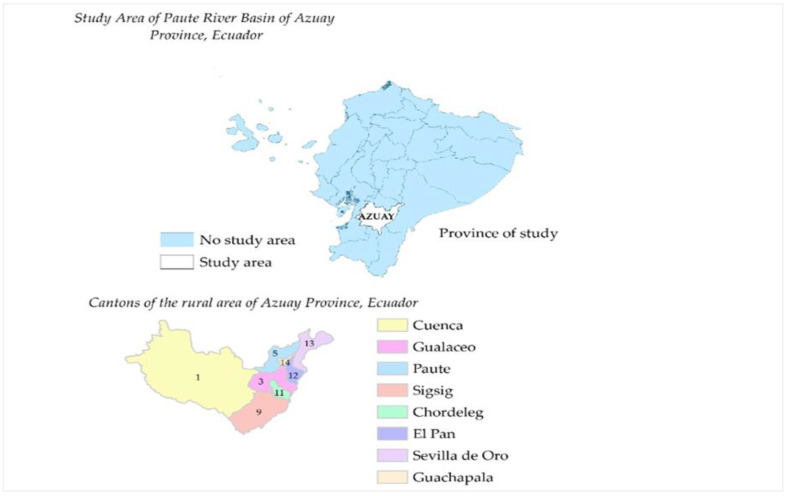
Location of the study area in the Paute River Basin, Azuay Province, Ecuador. Source: Authors’ own elaboration from the database.

The population under study is located in the rural area of the Paute River Basin in the province of Azuay, Ecuador. This area of study is important given the social, economic, demographic, and climate-change context experienced in recent years. Particularly, the rural area of study has faced in recent years drought, floods, landslides, among other natural disasters [6], which have caused effects on the social and economic field, in addition to having an influence on dietary diversity of the general population. More specifically, the dietary diversification survey was carried out in the following rural cantons: Cuenca, Gualaceo, Paute, Sigsig, Chordeleg, EI Pan, Sevilla de Oro, and Guachapala (Figure 1). 

### 2.2. Data Collection and Methods

The sample size was determined based on the total number of occupied private housing by type of housing, according to the last 2010 census conducted by National Statistics and Census Institute of Ecuador (INEC) [30], this ensures the representativeness of the sample in terms of population characteristics and economic and social variables. A stratified random sampling method with proportional allocation was applied. The data was obtained through a survey, with a sample size of 383 households. Furthermore, the data, collected at the household level, were from primary and cross-sectional sources. The pilot test of the survey was performed before the final survey. All surveys were undertaken in person and in accordance with consent for each participant, before being surveyed they received appropriate information about the research, in order to avoid conflicts between parties. The survey was applied to the head of the household or a member of the household present on the day of the survey. The final survey was carried out from March to May 2019. The surveys were distributed on different periods in order to ensure that every household had the same probability of being surveyed. It was not necessary to have assistance by translators from the area because the survey questionnaire of the dietary diversity was according to the context of study and in the Spanish language between both parties.

Therefore, the respondents thus gave their consent to participate in the research through an informed consent form in which their full names were requested along with their signature. The review, evaluation, and ethical approval for this research was granted by the Committee on Bioethics in Health Area Research (COBIAS) of the University of Cuenca, Ecuador. In addition, it is important to mention that the database of respondents will not be made public, to ensure the well-being and protection of the rights of participants. 

### 2.3. Questionnaire 

The survey questionnaire to build the HDDS indicator was based on the food groups proposed by Food and Nutrition Technical Assistance Project (FANTA) and the questionnaire was used at the household level according to the FANTA Household Dietary Diversity Score Indicator Guide. The socioeconomic and the agriculture questionnaire provides information about the characteristics of the rural households; as well as, provides information about the land and crop production. Finally, the program statistics STATA 14 was used for data analysis and inferences.

### 2.4. Dietary Diversity Indicator: HDDS

Dietary diversity is measured through a dietary diversity score, namely the HDDS developed by FANTA [31]. The dietary diversity score counts the number of different food groups consumed by the household over a certain period of time [32].

The HDDS indicator is expressed as:

(1) HDDS (0−12)=∑(A + B + C + D + E + F + G + H + I + J + K + L)

The HDDS indicator is calculated using 12 food groups over a recall period of 7 days: *A*. cereals; *B*. roots and tubers; *C*. fruits; *D*. sugar/honey; *E*. eggs; *F*. legumes or grains; *G*. vegetables; *H*. oils/fats; *I*. milk and dairy products; *J*. meats; *K*. miscellaneous; *L*. fish and seafood. The value of this indicator ranges from 0 to 12. The values for *A* to *L* can be “0” or “1”. The HDDS allows us to know the economic capacity of a household to access a variety of foods and has become the most commonly used indicator to measure the economic access of households to food [33,34]. 

### 2.5. Poisson Regression Model

The Poisson regression model is a generalized linear model (GLM) that meets the classical assumptions with only one exception, the distribution. The dependent variable assumes the Poisson distribution [35]; regardless of whether the distribution is maintained or not, asymptotically normal and consistent estimators of $B_{k}$ are obtained. 

The GLM is written as:

(2) $$g\left[E(y|x_{1},\;x_{2},\;\ldots ,x_{k})\;\right]=\;\beta_{0}\;+\;\beta^{T}x_{k}\;;\;\;\;y|x_{k}\;∼\;D\left(\theta \right),$$

where $\beta_{0}$ is the intersection term, β is a vector of coefficients, g (·) is a link function, and D(θ) is a distribution in the exponential family with one parameter θ. The Poisson regression model assumes a Poisson distribution P(θ) under an error structure and a logarithmic function as the linkage function [36]. 

The Poisson distribution has the property of robustness and is completely determined by its average $E(y|x_{k})$. This distribution, which is the basis of the Poisson regression model, allows us to find conditional probabilities for any value of the explanatory variables [37]. Then, the Poisson regression model is expressed as Equation (3):

(3) $$log\;\left[E\left(y|x_{1},\;x_{2},\;\ldots ,x_{k}\right)\right]=\;\beta_{0}+\beta_{1}x_{1}+\;\ldots +\beta_{k}x_{k};\;\;\;\;\;\;y|x_{k}\;∼\;P\left(\theta \right).$$

In particular, the expected value is expressed as an exponential function (Equation (3)), and the mean is equal to the variance (Equation (4)) [37]:

(4) $$E\left(y|x_{1},\;x_{2},\;\ldots ,x_{k}\right)=\;exp\left(\beta_{0}+\beta_{1}x_{1}+\;\ldots +\beta_{k}x_{k}\right),$$

(5) $$\mu =E\left(y|x_{k}\right)=\;Var\left(y|x_{k}\right)=\;exp\left(\beta_{0}+\;\beta^{T}x\right).$$

The probability density function (pdf) of the Poisson distribution is given by [38]:

(6) $$f\left(y\right)=\;\frac{\mu^{h}e^{-\mu }}{h!},\;\;h\;=\;0,\;1,\;2,\ldots ,$$

where f(y) is the probability that the variable *y* takes non-negative integer values (0, 1, 2,... *n*) and h! denotes a factorial. The dependent variable is a discrete-count variable, which can take non-negative integer values. Therefore, a Poisson regression model is more appropriate than a linear regression model.

The empirical application of the Poisson regression model is described in the following equation:

(7) $$g\left(y\right)=\log\left[E\left(y|x_{k}\right)\right]=\;\beta_{0}+\beta_{1}x_{1}+\beta_{2}x_{2}\;\ldots +\beta_{k}x_{k}+u_{i},$$

where y is the HDDS, a count dependent variable; $\beta_{0}$ is the intercept; $\beta_{1},\;\beta_{2},\;\ldots \beta_{k}$ are vectors of unknown parameters to be estimated; $x_{k}$ is a vector of explanatory variables of household i; $u_{i}$ is a robust standard error term. Explanatory variables include demographic, socioeconomic, and social characteristics (household size, age, sex, level of education of the head of household, and land area, among others). The factors selected as determinants of the HDDS were housing size, household size, age of the head of household (years), sex of the head of household, level of education of the head of household, marital status of the head of household (the marital status of the head of household was registered according to the options: single, married, widowed, divorced, consensual union, separated, and single mother), area of cultivated land (ha), total expenditure per capita (USD weekly), and food expenditure per capita (USD weekly). Therefore, nine explanatory variables were selected for the estimation of the model. The parameters and values chosen to rule that an association is or is not statistically significant was realized according the significance levels: * *p* < 0.10, ** *p* < 0.05, *** *p* < 0.01. The model parameter vector is estimated through the maximum likelihood method (MLE).

## 3. Results

**Figure 2:**
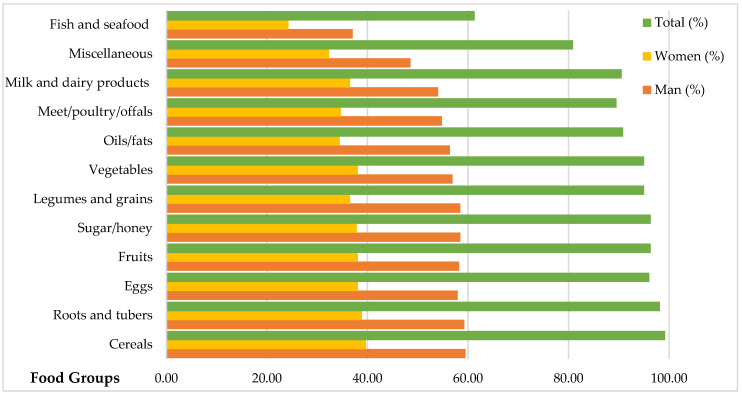
Total percentage of households that consumed each food group in 2019 and percentage broken down by the sex of the head of household. Note: *n* = 383 households. Source: Author’s own elaboration from surveys, 2019.

This section presents a descriptive and econometric analysis of the variables used in this research: socioeconomic, demographic, and food security indicator (Table 1 and Table 2). Similarly, Figure 2 shows the percentage of households, by sex of the head of household that consumed each food group in 2019. Finally, for the estimation of the robust Poisson regression model, we used a sample of 347 households, representing approximately 90% of the original sample (Table 2).

### 3.1. Descriptive Statistics 

Table 1 shows descriptive statistics for the socioeconomic and demographic variables used in this study. In the study area, housing size has a total of 4.90 rooms, on average, and a household is made up of 3.84 members, on average. Households of up to five members constitute 84.60% of those surveyed.

Regarding general expenses, on average, the total and weekly food expenditure per capita of households is approximately USD 17.00 and USD 12.00, respectively. Food security is low at the household level: only about 28% of households are food secure. In terms of agriculture, households have an average of 0.11 ha of cultivated land. That is, the occupied area of the land for crops is below 1 ha. In addition, 85% of households sow crops, and the crops planted, on average, are of approximately three types. 

Of the heads of household, 40% are women while 60% are men. The average age of the head of household is 47 years, and more than 50% are between 18 and 47 years old. The level of education of heads of household is low (7.83% with no education and 67.36% with primary education); furthermore, in most households, the head of the household is married (61.88%). Finally, in order to understand the dietary diversity of the respondents, the Household Dietary Diversity Score (HDDS) was calculated. The average HDDS is 10.89 foods, on a scale of 0 to 12 food groups.

The survey questionnaire is based on 12 food groups: cereals; roots and tubers; fruits; sugar/honey; eggs; legumes or grains; vegetables; oils/fats; milk and dairy products; meats; miscellaneous; fish and shellfish (see Appendix A Table A1). The details of the food groups consumed are illustrated in Figure 2. The results show that the foods most consumed are cereals, roots and tubers, eggs, sugar/honey, and fruits. More than 90% of households consume cereals. Furthermore, relatively more households with a head of household identified as male reported eating cereals. According to the total number of households and the sex of the head of the household, the least-consumed food category is fish and seafood (see Figure 2).

### 3.2. Econometric Estimation Analysis

The results of the econometric estimation are summarized in Table 2. The factors determining the HDDS were modeled using the Poisson regression model. Poisson regression allows for the modeling of count data—in this case, the dietary diversity score (which ranges from 0 to 12). In addition, when modeling the count data, this model assumes that the results are Poisson-distributed. In this research, prior to the choice of the most appropriate model of the factors that influence dietary diversity, it was necessary to compare the estimates of Poisson regressions and ordinary minimum squares (MCO) to ensure the robustness of the results. Indeed, two modeling approaches were used: Poisson and MCO, in order to ensure robust and reliable results. The ordinary least squares (OLS) regression was also estimated to validate the study findings. Comparing results from the two models is important because originally Poisson regression model was used when modeling count data. The set of explanatory variables under consideration were tested in both models and then the final model was determined. Therefore, the determinants of the dietary diversity score were estimated using OLS regression to test the robustness of the results. The coefficients were more significant in the Poisson model, and this model was selected according to the likelihood test and the Wald test. However, as the significant factors did not change that much compared to the OLS model, so the Poisson results are reliable, robust, and estimated with robust standard errors.

Table 2 shows the coefficients of the different factors that determine the HDDS at the household level, their robust standard errors, and the limits of the confidence intervals. The coefficients can be interpreted as elasticities or semielasticities, representing a percentage change in the dietary diversity score when the explanatory variable changes by one unit. The positive and significant coefficients in column (2) suggest that the HDDS of households increases with a change in one of these explanatory variables (Table 2). The results of the robust model show that the housing size, household size, the level of education of the head of the household, the marital status of the head of the household, the area of cultivated land (ha), and the per capita food expenditure are statistically significant variables and important factors determining the HDDS. 

The housing size has a positive and significant effect with respect to the HDDS (*p* < 0.1). The household size variable also has a *p* value below 0.05 and is therefore significantly associated to HDDS. The household size variable has a positive coefficient, meaning that when the number of household members is large the HDDS increases. The coefficient of the education of the head of household variable, at the secondary or higher level, is positive and significantly influences the dietary diversity score of households (*p* < 0.1). Therefore, a high level of education of the head of the household is associated with an increased probability of having a higher HDDS. The marital status of the head of household shows a statistically significant association with the HDDS, the categories divorced (*p* < 0.01), consensual union (*p* < 0.05), and separated (*p* < 0.05) have a positive effect on the HDDS. The coefficient of the cultivated land area variable is positive and is statistically associated with the HDDS (*p* <0.01). In other words, having access to more land increases the dietary diversity score and probably the ability to produce a greater variety of crops. Similarly, the ratio of food expenditure per capita is statistically significant and is positively associated with the HDDS (*p* < 0.01). 

The model results also indicate that age of the head of household, sex of the head of household, and total per capita household expenditure are nonsignificant factors in determining the HDDS (Table 2). This means that there is no statistically significant association between these variables and the HDDS. In this case, only the effect of the signs of the variables in question can be interpreted. The age of the head of household presents a negative sign, implying that having an older head of household is associated with a decrease in the HDDS. The head of household’s sex variable shows a positive coefficient, which means that when households are headed by women the HDDS tends to be high. Finally, the total per capita household expenditure variable also shows a positive sign, indicating that high per capita household expenditure is associated with a high HDDS.

## 4. Discussion

The purpose of this research was to analyze the factors that determine the Household Dietary Diversity Score (HDDS) in the rural area of the Paute River Basin, Azuay Province, Ecuador, and compare the results to those of other studies [40,41,42,43].

In the global literature and that of Latin America (LA), various studies focus on dietary diversity and the HDDS [5,32,44,45,46,47], presenting important findings that can be compared with the results of the present study. Within the Ecuadorian literature, important studies of dietary diversity include an analysis of nutritional diversity in the province of Imbabura [48] and of malnutrition in Ecuadorian mothers [49], where food is available and can meet the caloric and nutritional needs in the home. Up until now, few studies have investigated and analyzed the determinants of the dietary diversity at the regional or local level, as the country is undergoing a nutrition transition and little is known about household-level food insecurity in middle-income where about a quarter of the population lives in poverty [10,50,51,52]. In an attempt to help fill this gap, the present study takes and in-depth look at a dietary diversity score in the rural area of the Paute River Basin, Azuay Province, Ecuador.

In most cases and according to the literature, food security is low in rural areas (< 34%), and improving food security is a persistent development challenge of global political concern [53,54]. According to the results found here, the food security indicator is also low (28%) in the study area. However, agriculture is one of the most important economic activities in rural areas and can influence the quality of the diets of households in rural areas [55]. According to the results of this study, 85% of households sow a variety of crops, with below 1 ha of land cultivated with the maximum number of crops planted being 12. In this research, the average area of land cultivated by one household in this study is below 1 ha. The area of land to be cultivated is an important factor for dietary diversity, however, and households that own more agricultural land tend to enjoy higher dietary diversity [5,56]. Access to different foods is a good technique to estimate the diversity of the diets of rural households, taking into account that each food has its own importance among all food groups, including staple foods [56,57]. It is crucial for households to not only consume adequate amounts of food but also safe and diversified foods. In Ecuador, at the national level, it is estimated that around 30% of the population has an excessive consumption of carbohydrates [58,59].

According to our results, most households are highly dependent on staple crops such as cereals and roots and tubers due to their low cost [57]. The food group least consumed in households is fish and shellfish, which is due to lack of income [43,60,61,62,63,64]. The current findings show that rural households have a high dietary diversity score, and the higher score could probably improve the quality of the diet. In general, a preference for diverse foods could motivate people to seek a more diversified diet [15,65]. Among the food groups, the consumption of fresh and natural foods, namely fruits, vegetables, and foods containing vitamins and minerals are important, since its deficiency leads to households being more susceptible to having food security problems [59,66]. In this context, participation in agriculture is an important strategy to maintain food diversity in the short and long term, as well as to eradicate hunger and provide security from environmental, climate, economic, and social change [57]. In this research, we analyze the rural households in their own context. This will lead to future research regarding what is the grade of participation in agriculture necessary for maintaining food diversity in the communities.

Dietary diversity provides information on household access to a variety of foods [67], and the Household Dietary Diversity Score (HDDS) is intended to reflect a household’s economic ability to access a variety of foods [32]. In other studies, relationships between the HDDS and the socioeconomic characteristics of the households have been captured, including age, sex, level of education of the head of household, employment status or income, food expenditure, size of agricultural land, training received regarding food preparation and human nutrition, among others [9,55,68,69]. It is clear that the crisis generated by the COVID-19 pandemic is having and will continue to have consequences on household diets. According to the International Monetary Fund, food prices will by 1.56% in 2020 and then increase by 0.56% in 2021 [70]. Thus, in terms of food that is not produced by rural households themselves, it can be said that access will have increased at least during 2019, but income from the sale of products will have decreased.

On the other hand, an interesting result of the current study is that there is a positive relationship between the HDDS and the housing size. Other studies have found a relationship between house or farm size with respect to the HDDS [23,32,42]. The household size (number of members) was a significant variable with respect to the HDDS. This variable often increases the food demands for adults in the household, and in some cases can increase dietary diversity [71]. The results also show that the level of education of the head of the household contributes to the improvement of the HDDS. Several studies have shown a positive association between level of education and dietary diversity [5,9,42,68,72]. This means that education has an impact on household nutritional knowledge and the skills to conceptualize and use messages promoting nutrition, consequently contributing to greater dietary diversity [41]. Thus, the progress of nutrition education depends on the quality and innovation of an educational program, the acceptability of the message, local understanding, and behavioral change [57]. For this reason, it is important to support programs providing information about food and its preparation, consumption, and utilization to address food and nutrition challenges within a community [9]. Similarly, the marital status of the head of the household, namely being divorced, in a consensual union, or separated, has a significant positive effect on the HDDS, and in other studies, similar results have been found [45,67]. The area of cultivated land is an important factor that also contributes to improving the dietary diversity of households, and it is important in terms of food production and food diversity. Generally, having access to more land increases the capacity to produce a greater variety of crops [9,57]. The area of land cultivated is an important factor for dietary diversity, and households with agricultural land tend to have a higher HDDS [9]. Therefore, maintaining multiple crops in one’s fields helps to ensure food security and food diversity [57]. In addition, results also indicate that per capita food expenditure contributes to improving the HDDS. The coefficient of this variable, being statistically significant and positively associated with the HDDS, shows that the purchasing of food is important to achieve greater household dietary diversity [55], to meet the basic food needs of households.

In this study, determinants that were not significant with respect to the HDDS included age and sex of the head of household and total per capita expenditures. In a study by Isabirye et al. [67], some variables that were not significant factors in determining dietary diversity included age, education, work status, and household size, among others. Finally, it is important to mention that the results found here in regard to significant and nonsignificant variables are similar to the findings reported by studies of other rural areas [9,19,24,67,71,73]. These results have allowed us to know the context of other places and in this way to be able to compare with our results.

## 5. Conclusions

Food diversity is mainly determined by access to food. The modern lifestyle has affected the quality of the diet, due to the increase in the consumption of foods rich in saturated fat, calories, salt, sugar, and little physical activity; making overweight and obesity more visible, which has caused an increase in Non-Communicable Diseases (NCDs) associated with food. For this reason, studying the different factors involved in dietary diversity can help improve food security and thus reduce malnutrition.

The Household Dietary Diversity Score (HDDS) is an indicator used to measure access to food, assess dietary patterns, and study changes in diet over a short period [31]. However, cut-off points and reference time periods vary, making comparisons between studies difficult [74]. In this study, the determination of HDDS as an indicator of economic access to food among households, enabled the evaluation of dietary diversity in this population and the assessment of the variety of food groups consumed. The average HDDS was 10.89 foods, on a scale of 0–12 food groups. The most consumed food was cereals, and the least consumed was fish and seafood. The results of the robust model mainly show that the marital status of the head of the household, the area of cultivated land (ha), and the per capita food expenditure are variables that increase the dietary diversity score (HDDS).

Thus, access to land is an important issue that must remain on the agenda of public agricultural policies in Ecuador, as rural families in a situation of food insecurity fundamentally depend on agriculture for their livelihoods. For this reason, improving the land-access situation of heads of households can have a positive impact on food access, helping to make diets more diverse.

The data from this study revealed that households spent a large part of their spending on food. Therefore, the purchase of food is important to achieve greater dietary diversity in households. However, these families could be vulnerable to drastic changes in food prices. In addition, it is crucial that the government emphasizes the need to incorporate policies, plans, and programs that contribute to the food security of families. Such as, for example, economic policies and programs such as social protection, assistance for employment and housing, tax relief measures, among others.

On the other hand, education has positive effects in this study, because there is a greater probability that households have a higher HDDS, playing an important role in dietary diversity and therefore in household food security. Therefore, access to education can ensure higher income, which means greater access to food. In addition, it is recommended that, in educational plans at all levels, content on food and nutrition education is incorporated, so that people can eat in a conscious and responsible way—from planning the food they are going to buy, preparing, the consumption, reuse and disposal of food—with the aim that these people have as a result a healthy diet or improvements in it, and of promoting the prevention of risk behaviors associated with non-communicable diseases, derived from unhealthy eating habits and lifestyles.

It is recommended that, in order for there to be improvements in food diversity in households, both locally and nationally, the Food Sovereignty Law approved in Ecuador since 2009 should be put into practice, which “aims to establish the mechanisms through which the State fulfills its obligation and strategic objective of guaranteeing individuals, communities and peoples the self-sufficiency of healthy, nutritious and culturally appropriate food on a permanent basis”.

It is worth mentioning some of the limitations of this research. First, the sample used in this study is not representative of all rural areas in Ecuador. Therefore, the results may not be comparable if other rural areas have a different socioeconomic structure. It would be of great interest to conduct similar research with a larger number of participants and in different regions of Ecuador to consolidate our preliminary findings. It would also be beneficial to include an investigation of the per capita availability of calories in households, as in the current study it was only possible to measure access to food and levels of dietary diversity. No comparisons were made with nutrient requirements in different contexts, for example, no analysis was made of the composition of the food consumed by households. Moreover, the applied statistical analysis only provides an assessment of the effect—positive or negative—of dietary diversity and does not provide an economic estimate. Despite these limitations, the main findings of this research form an initial baseline, providing a greater understanding of food diversity in the rural area of the Paute River basin in the province Azuay, Ecuador. In addition, the results of this research may serve as support to policymakers in targeting and monitoring efforts to improve the food security, especially in rural households. To increase the precision of food security measurements, future research should focus on the analysis of multiple indicators, taking into consideration COVID-19, with an emphasis on rural areas, and targeting communities at risk. To increase the precision of food security measures, future research should focus on the analysis of multiple indicators, considering COVID-19, with an emphasis on rural areas and targeting communities at risk. For example, it is recommended to conduct a Food Safety assessment during COVID-19 and compare the data with the results of this investigation. In addition, it would be key to measure the short-term capacity of households to cope with the health crisis, asking how many times in the previous week, they had applied any of the five coping strategies due to lack of sufficient food and/or resources (strategies of coping: of consumption, of reducing the size of the portion of meals, of reducing the quantities of meals, and restricting the consumption of adults to satisfy the needs of minors).

## Figures and Tables

**Table 1:** Socioeconomic and demographic characteristics.

Variables	Observations	Mean/Percentage (%)	Standard Deviation	Min.	Max.
	*n* = 383				
**Housing and Household Size**					
Housing size (number of rooms)	382	4.90	1.59	1	10
Household size (number of household members)	383	3.84	1.65	1	10
Family size					
≤5 members	324	84.60%			
6–8 members	55	14.36%			
≥8	4	1.04%			
**General Expenditures**					
Total expenditure per capita (weekly)	348	16.76	14.03	0.86	116.67
Food expenditure per capita (weekly)	359	12.10	8.87	1	60
**Food Security** (Constructed from the questions on the Latin American and Caribbean Food Security Scale (ELCSA) questionnaire) [39]					
Indicator of food security in rural households	383	28.46%			
**Agriculture**					
Area of cultivated land (ha)	383	0.11	1.11	0	20
Sown crops	323	85.00%			
Unsown crops	57	15.00%			
Total crops	383.00	3.19	2.183	0	12
**Sex of the Head of Household**					
Female	152	39.69%			
Male	231	60.31%			
**Age of the Head of Household**	383	47.20	16.25	18	86
Age categories (years)					
18–47	198	51.70%			
48–60	97	25.33%			
≥61	88	22.98%			
**Educational Level of the Head of Household**					
Uneducated	30	7.83%			
Primary	258	67.36%			
Secondary or higher	95	24.80%			
**Marital Status of the Head of Household**					
Single	50	13.05%			
Married	237	61.88%			
Widowed	36	9.40%			
Divorced	9	2.35%			
Consensual union	28	7.31%			
Separated	9	2.35%			
Single mother	14	3.66%			
**Dietary Diversity**					
Household Dietary Diversity Score (HDDS)	383	10.89	1.28	4	12

Source: Author’s own elaboration from surveys, 2019.

**Table 2:** Results of the robust Poisson regression analysis showing the factors that determine the HDDS in rural households.

HDDS	Coefficients	Robust Standard Error	*p*-Value	Confidence Interval (95%)	
				Lower	Upper
Housing size (number of rooms)	0.007	0.003	0.057 *	0.000	0.013
Household size (number of members)	0.005	0.005	0.013 **	0.003	0.022
Age of the head of household (years)	−0.001	0.000	0.206	−0.001	0.000
Sex of the head of household (male = 0, female = 1)					
*Female*	0.001	0.015	0.959	−0.029	0.031
Level of education of the head of household (uneducated = 0, primary = 1, secondary or higher = 2)					
*Primary*	0.030	0.033	0.362	−0.035	0.095
*Secondary or higher*	0.062	0.034	0.072 *	−0.005	0.129
Marital status of the head of household (single = 0, married = 1, widow(er) = 2, divorced = 3, consensual union = 4, separated = 5, single mother = 6)					
*Married*	0.003	0.020	0.867	−0.035	0.042
*Widow(er)*	−0.039	0.029	0.173	−0.095	0.017
*Divorced*	0.077	0.026	0.003 ***	0.026	0.127
*Consensual union*	0.051	0.023	0.022 **	0.007	0.096
*Separated*	0.057	0.026	0.029 **	0.006	0.109
*Single mother*	−0.004	0.035	0.906	−0.073	0.065
Area of cultivated land (ha)	0.006	0.002	0.001 ***	0.003	0.010
Total expenditure per capita (USD weekly)	0.001	0.001	0.302	−0.001	0.002
Food expenditure per capita (USD weekly)	0.003	0.001	0.001 ***	0.001	0.006
_cons	2.235	0.041	0.000	2.155	2.315
Log pseudolikelihood=	−756.2957				
Number of obs.=	347.0000				
Wald chi^2^ (15)=	110.7300				
Prob > chi^2^=	0.0000				
Pseudo R^2^=	0.0076				

Note: Significance levels: * *p* < 0.10, ** *p* < 0.05, *** *p* < 0.01. HDDS: Household Dietary Diversity Score. Source: Authors’ own elaboration from surveys, 2019.

## Data Availability

The data are not publicly available due to privacy and ethical.

## Appendix A

**Table A1:** Food group classification.

Food Group	Description
1. Cereals	Rice, noodles, bread, corn, beans, others
2. Roots and tubers	Potato, cassava, others
3. Legumes and grains	Beans, chickpeas, broad beans, peas, others
4. Fish and seafood	Fish, canned tuna, other shellfish
5. Eggs	Eggs by purchase or own production
6. Milk and dairy products	Milk, yogurt, cheese, other dairy products (excludes butter/margarine)
7. Vegetables	Carrot, spinach, turnip, cabbage, cauliflower, broccoli, onion, tomato, cucumber, radish, others
8. Fruits	Apple, banana, pear, peach, mango, papaya, melon, orange, lemon, mandarin orange, others
9. Sugar/honey and other sugars	Sugar, honey, jam; panela, cakes, cookies, sodas and other sugary drinks
10. Miscellaneous	Drinks: tea, coffee, cocoa; seasonings: salt, garlic, baking powder
11. Meat, poultry, offal	Beef, chicken, pork, others; liver, kidney, heart, others
12. Oils and fats	Butter, vegetable oil, palm oil, margarine, other fats

Source: Authors’ own elaboration from surveys, 2019.

## References

[1] FAO, IFAD, UNICEF, WFP, WHO (2020). The State of Food Security and Nutrition in the World 2020.

[2] FAO, PAHO, WFP, UNICEF (2019). Regional Overview of Food Security and Nutrition in Latin America and the Caribbean 2019.

[3] United Nations (2020). Policy Brief: The Impact of COVID-19 on Latin America and the Caribbean.

[4] World Food Programme (WFP) Hunger Map 2020 Chronic Hunger. https://www.wfp.org/publications/hunger-map-2020.

[5] Chegere M.J., Stage J. (2020). Agricultural production diversity, dietary diversity and nutritional status: Panel data evidence from Tanzania. World Dev..

[6] Armstrong B., Hepworth A.D., Black M.M. (2020). Hunger in the household: Food insecurity and associations with maternal eating and toddler feeding. Pediatr. Obes..

[7] Lin I.-H., Van Duong T., Nien S.-W., Tseng I.-H., Wang H.-H., Chiang Y.-J., Chen C.-Y., Wong T.-C. (2020). Dietary diversity score: Implications for obesity prevention and nutrient adequacy in renal transplant recipients. Int. J. Environ. Res. Public Health.

[8] Singh J.K., Acharya D., Gautam S., Adhikari M., Park J.-H., Yoo S.-J., Lee K. (2019). Socio-demographic and diet-related factors associated with insufficient fruit and vegetable consumption among adolescent girls in rural communities of Southern Nepal. Int. J. Environ. Res. Public Health.

[9] Ochieng J., Afari-Sefa V., Lukumay P.J., Dubois T. (2017). Determinants of dietary diversity and the potential role of men in improving household nutrition in Tanzania. PLoS ONE.

[10] Symonds A. (2017). Early Child Development Centers Positively Impact Food Security, Dietary Diversity, Growth, and Developmental Outcomes: An Analysis of Two Early Child Development Centers in Estancia, El Salvador. Ph.D. Thesis.

[11] Deaconu A., Mercille G., Batal M. (2019). The agroecological farmer’s pathways from agriculture to nutrition: A practice-based case from ecuador’s highlands. Ecol. Food Nutr..

[12] Alam A., Chowdhury M., Dibley M.J., Raynes-Greenow C. (2020). How can we improve the consumption of a nutritionally balanced maternal diet in rural Bangladesh? The key elements of the “Balanced Plate” Intervention. Int. J. Environ. Res. Public Health.

[13] Abeywickrama H.M., Wimalasiri K.M.S., Koyama Y., Uchiyama M., Shimizu U., Chandrajith R., Nanayakkara N. (2020). Assessment of nutritional status and dietary pattern of a rural adult population in Dry Zone, Sri Lanka. Int. J. Environ. Res. Public Health.

[14] Herforth A., Jones A., Andersen P.P. (2012). Prioritizing Nutrition in Agriculture and Rural Development: Guiding Principles for Operational Investments.

[15] Fanzo J., Hunter D., Borelli T., Mattei F. (2013). Diversifying Food and Diets: Using Agricultural Biodiversity to Improve Nutrition and Health.

[16] Ruel M.T. (2003). Operationalizing dietary diversity: A review of measurement issues and research priorities. J. Nutr..

[17] Berti P.R., Jones A.D., Fanzo J., Hunter D., Borelli T., Mattei F. (2013). Biodiversity’s contribution to dietary diversity: Magnitude, meaning and measurement. Diversifying Food and Diets: Using Agricultural Biodiversity to Improve Nutrition and Health.

[18] Contreras Díaz J., Paredes M., Turbay S. (2017). Agroecological short circuits of marketing in Ecuador. Idesia.

[19] Huluka A.T., Wondimagegnhu B.A., Yildiz F. (2019). Determinants of household dietary diversity in the Yayo biosphere reserve of Ethiopia: An empirical analysis using sustainable livelihood framework. Cogent Food Agric..

[20] Kennedy G., Ballard T., Dop M. (2013). Guía Para Medir la Diversidad Alimentaria a Nivel Individual y del Hogar.

[21] Rodríguez A.G. (2017). Agenda 2030 para el Desarrollo Sostenible y Sistemas Alimentarios Sostenibles. Una Propuesta para la Formulación de Políticas Integradoras.

[22] Vaitla B., Coates J., Glaeser L., Hillbruner C., Biswal P., Maxwell D. (2017). The measurement of household food security: Correlation and latent variable analysis of alternative indicators in a large multi-country dataset. Food Policy.

[23] Koppmair S., Kassie M., Qaim M. (2017). Farm production, market access and dietary diversity in Malawi. Public Health Nutr..

[24] Jebessa G., Sima A., Wondimagegnhu B. (2019). Determinants of household dietary diversity in Bangladesh. Ethiop. J. Sci. Technol..

[25] Oyarzun P.J., Borja R.M., Sherwood S., Parra V. (2013). Making sense of agrobiodiversity, diet, and intensification of smallholder family farming in the Highland Andes of Ecuador. Ecol. Food Nutr..

[26] Walrod J., Seccareccia E., Sarmiento I., Pimentel J.P., Misra S., Morales J., Doucet A., Andersson N. (2018). Community factors associated with stunting, overweight and food insecurity: A community-based mixed-method study in four Andean indigenous communities in Ecuador. BMJ Open.

[27] De Janvry A., Sadoulet E. (2006). Progress in the modeling of rural households’ behavior under market failures. Poverty, Inequality, and Development: Essays in Honor of Erik Thorbecke.

[28] Penafiel D., Cevallos-Valdiviezo H., Espinel R., Van Damme P. (2019). Local traditional foods contribute to diversity and species richness of rural women’s diet in Ecuador. Public Health Nutr..

[29] Fanzo J., Hunter D., Borelli T., Mattei F. (2013). Agricultural biodiversity, diverse diets and improving nutrition. Diversifying Food and Diets: Using Agricultural Biodiversity to Improve Nutrition and Health.

[30] INEC Base de Datos—Censo de Población y Vivienda. https://www.ecuadorencifras.gob.ec/base-de-datos-censo-de-poblacion-y-vivienda/.

[31] Swindale A., Bilinsky P. (2006). Household Dietary Diversity Score (HDDS) for Measurement of Household Food Access: Indicator Guide.

[32] Sibhatu K.T., Qaim M. (2018). Farm production diversity and dietary quality: Linkages and measurement issues. Food Secur..

[33] Kennedy G., Ballard T., Dop M. (2013). Guidelines for Measuring Household and Individual Dietary Diversity.

[34] Headey D.D., Ecker O. (2013). Rethinking the measurement of food security: From first principles to best practice. Food Secur..

[35] Cupal M., Deev O., Linnertova D. (2015). The poisson regression analysis for occurrence of floods. Procedia Econ. Financ..

[36] Takahashi A., Kurosawa T. (2016). Regression correlation coefficient for a Poisson regression model. Comput. Stat. Data Anal..

[37] Wooldridge J.M. (2013). Limited dependent variable models and sample selection corrections. Introductory Econometrics. A Modern Approach.

[38] Gujarati D.N., Porter D.C. (2009). Qualitative response regression models. Basic Econometrics.

[39] FAO (2012). Escala Latinoamericana y Caribeña de Seguridad Alimentaria (ELCSA). Manual de Uso y Aplicación.

[40] Drammeh W., Njie B., Hamid N.A., Rohana A.J. (2020). Determinants of dietary diversity among households in central river region south, the Gambia. Curr. Res. Nutr. Food Sci. J..

[41] Rajendran S., Afari-Sefa V., Shee A., Bocher T., Bekunda M., Dominick I., Lukumay P.J. (2017). Does crop diversity contribute to dietary diversity? Evidence from integration of vegetables into maize-based farming systems. Agric. Food Secur..

[42] Morseth M.S., Grewal N.K., Kaasa I.S., Hatloy A., Barikmo I., Henjum S. (2017). Dietary diversity is related to socioeconomic status among adult Saharawi refugees living in Algeria. BMC Public Health.

[43] Agrawal S., Kim R., Gausman J., Sharma S., Sankar R., Joe W., Subramanian S.V. (2019). Socio-economic patterning of food consumption and dietary diversity among Indian children: Evidence from NFHS-4. Eur. J. Clin. Nutr..

[44] Cordero-Ahiman O., Santellano-Estrada E., Garrido A. (2017). Dietary diversity in rural households: The case of indigenous communities in Sierra Tarahumara, Mexico. J. Food Nutr. Res..

[45] Amugsi D.A., Dimbuene Z.T., Bakibinga P., Kimani-Murage E.W., Haregu T.N., Mberu B. (2016). Dietary diversity, socioeconomic status and maternal body mass index (BMI): Quantile regression analysis of nationally representative data from Ghana, Namibia and Sao Tome and Principe. BMJ Open.

[46] Mayén A.-L., Marques-Vidal P., Paccaud F., Bovet P., Stringhini S. (2014). Socioeconomic determinants of dietary patterns in low- and middle-income countries: A systematic review. Am. J. Clin. Nutr..

[47] Kiboi W., Kimiywe J., Chege P. (2017). Determinants of dietary diversity among pregnant women in Laikipia County, Kenya: A cross-sectional study. BMC Nutr..

[48] Melby C.L., Orozco F., Averett J., Muñoz F., Romero M.J., Barahona A. (2020). Agricultural food production diversity and dietary diversity among female small holder farmers in a region of the Ecuadorian Andes experiencing nutrition transition. Nutrients.

[49] Freire W.B., Waters W.F., Rivas-Mariño G., Belmont P. (2018). The double burden of chronic malnutrition and overweight and obesity in Ecuadorian mothers and children, 1986–2012. Nutr. Health.

[50] Irwin S., Flaherty M.S., Carolsfeld J. (2020). The contribution of small-scale, privately owned tropical aquaculture to food security and dietary diversity in Bolivia. Food Secur..

[51] Tiburcio E. (2016). Food Insecurity in San Pedro, Ecuador: The Association of Food Insecurity with Dietary Diversity and BMI. Master’s Thesis.

[52] Bernal Rivas J., Lorenzana Albert P. (2003). Dietary diversity and associated factors among beneficiaries of 77 child care centers: Central Regional, Venezuela. Arch. Latinoam. Nutr..

[53] Sunderland T., Powell B., Ickowitz A., Foli S., Pinedo-Vasquez M., Nasi R., Padoch C. (2013). Food Security and Nutrition: The Role of Forests.

[54] Niragira S., Ndimubandi J., Van Orshoven J. (2019). Income, time and labor nexus household food security in Burundi. Encyclopedia of Food Security and Sustainability.

[55] Jones A.D., Shrinivas A., Bezner-Kerr R. (2014). Farm production diversity is associated with greater household dietary diversity in Malawi: Findings from nationally representative data. Food Policy.

[56] Muthini D., Nzuma J., Qaim M. (2020). Subsistence production, markets, and dietary diversity in the Kenyan small farm sector. Food Policy.

[57] Powell B., Bezner Kerr R., Young S.L., Johns T. (2017). The determinants of dietary diversity and nutrition: Ethnonutrition knowledge of local people in the East Usambara Mountains, Tanzania. J. Ethnobiol. Ethnomed..

[58] Segovia J., Orellana M., Sarmiento J.P., Carchi D. (2020). The effects of taxing sugar-sweetened beverages in Ecuador: An analysis across different income and consumption groups. PLoS ONE.

[59] Freire W.B., Ramírez M.J., Belmont P., Mendieta M.J., Silva M.K., Romero N., Sáenz K., Piñeiros P., Gómez L.F., Monge R. (2013). RESUMEN EJECUTIVO. TOMO I. Encuesta Nacional de Salud y Nutrición del Ecuador.

[60] Cordero-Ahiman O.V., Vanegas J.L., Beltrán-Romero P., Quinde-Lituma M.E. (2020). Determinants of food insecurity in rural households: The case of the Paute river basin of Azuay Province, Ecuador. Sustainability.

[61] Fiese B.H., Gundersen C., Koester B., Jones B. (2016). Family chaos and lack of mealtime planning is associated with food insecurity in low income households. Econ. Hum. Biol..

[62] Ritzema R., Douxchamps S., Fraval S., Bolliger A., Hok L., Phengsavanh P., Long C., Hammond J., Van Wijk M. (2019). Household-level drivers of dietary diversity in transitioning agricultural systems: Evidence from the Greater Mekong Subregion. Agric. Syst..

[63] Vega-Macedo M., Shamah-Levy T., Peinador-Roldán R., Humarán I.M.-G., Melgar-Quiñonez H. (2014). Inseguridad alimentaria y variedad de la alimentación en hogares mexicanos con niños menores de cinco años. Salud Pública México.

[64] Legwegoh A.F., Hovorka A.J. (2013). Assessing food insecurity in Botswana: The case of Gaborone. Dev. Pract..

[65] Hawkes C. (2008). Dietary implications of supermarket development: A global perspective. Dev. Policy Rev..

[66] Instituto Nacional de Estadísticas y Censos (INEC) (2015). Encuesta Condiciones de Vida VI Ronda 2015.

[67] Isabirye N., Bukenya J.N., Nakafeero M., Ssekamatte T., Guwatudde D., Fawzi W. (2020). Dietary diversity and associated factors among adolescents in eastern Uganda: A cross-sectional study. BMC Public Health.

[68] Heim A., Paksi A. (2019). Low dietary diversity and its influencing factors among a San group in Namibia. BMC Res. Notes.

[69] Parappurathu S., Kumar A., Bantilan M.C.S., Joshi P.K. (2015). Food consumption patterns and dietary diversity in eastern India: Evidence from village level studies (VLS). Food Secur..

[70] International Monetary Fund (2020). World Economic Outlook: The Great Lockdown.

[71] Christian A.K., Marquis G.S., Colecraft E.K., Lartey A., Soueida R. (2019). Household food insecurity but not dietary diversity is associated with children’s mean micronutrient density adequacy in rural communities across Ghana. Nutrition.

[72] Torheim L.E., Ouattara F., Diarra M.M., Thiam F.D., Barikmo I., Hatløy A., Oshaug A. (2004). Nutrient adequacy and dietary diversity in rural Mali: Association and determinants. Eur. J. Clin. Nutr..

[73] Mbwana H.A., Kinabo J., Lambert C., Biesalski H.K. (2016). Determinants of household dietary practices in rural Tanzania: Implications for nutrition interventions. Cogent Food Agric..

[74] Carletto C., Zezza A., Banerjee R. (2013). Towards better measurement of household food security: Harmonizing indicators and the role of household surveys. Glob. Food Secur..

